# Arthropod‐Inspired Miniature Modular Vibration Direct‐Drive Robot With Untethered Locomotion

**DOI:** 10.1002/advs.77834

**Published:** 2026-09-16

**Authors:** Shijing Zhang, Ziteng Liu, Youwei Liu, Dehong Wang, Yingxiang Liu

**Affiliations:** ^1^ State Key Laboratory of Robotics and Systems Harbin Institute of Technology Harbin China

**Keywords:** bionic design, miniature robots, modular robots, untethered locomotion, vibration direct‐drive

## Abstract

Miniature vibration direct‐drive robots hold immense potential for missions in confined spaces, such as pipeline inspection and environmental detection. However, conventional fixed‐configuration designs are restricted to single functionality, failing to adapt to the multifaceted demands of diverse operational tasks. Here, we present an arthropod‐inspired untethered miniature modular vibration direct‐drive robot, which achieves on‐demand reconfiguration through standardized 19 mm × 32.5 mm × 18 mm driving units. Core innovations include tool‐less nested locking interfaces for rapid assembly; compliant silicone connectors designed to mitigate vibration‐induced wear and provide vibration isolation; chevron‐shaped feet optimizing vibration‐to‐locomotion conversion; and a highly integrated onboard control architecture enabling untethered operation. The robot reconfigures into quadruped, hexapod, octopod, and circular morphologies with stable performance. In the quadruped robot (58.4 g; 46 mm × 75 mm × 38.8 mm), it reaches a maximum linear speed of 580.74 mm/s and rotational speed of 16.64 rad/s. The octopod robot sustains 450 mm/s under a 60 g payload while traversing 2.8 mm obstacles, 30 mm gullies, and confined pipelines. This work breaks fixed‐configuration limitations, providing a generalizable paradigm for synergizing modularity and vibration‐driven efficiency at the centimeter scale, offering new insights for miniature robots, and a versatile platform for confined‐space operations.

## Graphical abstract

This work presents an arthropod‐inspired untethered miniature modular vibration direct‐drive robot. By utilizing standardized driving units and nested locking interfaces, on‐demand reconfiguration into quadruped, hexapod, octopod, and circular morphologies is achieved. The robot demonstrates exceptional locomotion performance, including high speed, significant payload capacity, and versatility in traversing complex confined environments like pipelines.

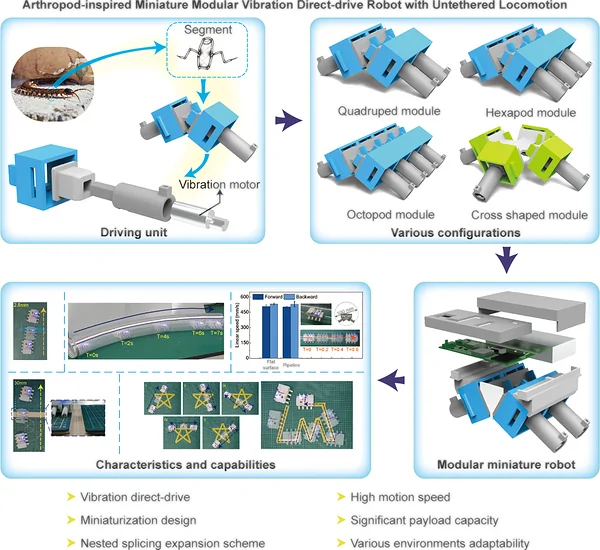

## 1 Introduction

Miniature robots feature core advantages of simple and compact structure, flexible and controllable motion, and outstanding environmental adaptability. They can operate efficiently in narrow spaces or complex unstructured scenarios inaccessible to humans and traditional large‐scale robots, while integrating low power consumption, high integration, and strong robustness, thus emerging as a research hotspot in the current robotics field [1, 2, 3, 4, 5, 6]. Vibration direct‐drive is one of the important driving methods for miniature robots [7, 8, 9, 10, 11, 12, 13, 14, 15, 16, 17, 18, 19, 20, 21, 22, 23, 24, 25, 26, 27, 28, 29, 30, 31]. In contrast to electromagnetic motor wheeled direct‐drive [32, 33], vibration direct‐drive offers simpler control strategies and lower excitation voltage requirements. For instance, Andrew T et al. developed the Harvard ambulatory micro robot with passive spines (HAMR‐VP), which adopts piezoelectric bimorph actuators and a flexible five‐bar hip joint structure, and achieves high‐efficiency integration based on printed circuit micro‐electro‐mechanical system (MEMS) technology and pop‐up assembly technology, with an overall weight of only 1.27 g. Utilizing the resonant characteristics of piezoelectric actuators, the robot generates elliptical motion trajectories at the legs by tuning the phase and amplitude of the driving signal, enabling adaptive motion at high and low speeds without additional steering mechanisms [7]. Li et al. proposed the agile quadruped piezoelectric robot (AQPR), which leverages the mode degeneracy principle to generate multi‐dimensional elliptical trajectories at the feet, enabling planar two‐dimensional motion without complex transmission components. With a dimension of only 30 × 30 × 14.3 mm^3^ and a weight of 6.9 g, this robot achieves a payload capacity of 30 times its own weight (200 g) by virtue of the high stiffness of its annular structure, a maximum linear motion speed of 255 mm/s, and a rotational speed of up to 1265°/s. Meanwhile, it realizes a linear resolution of 0.25 µm and a rotational resolution of 32.7 µrad via pulse signal control, and has been successfully applied in micro‐manipulation scenarios such as wafer defect detection and target recognition [8].

Common vibration direct‐drive actuators include those based on novel smart materials such as piezoelectric materials, shape memory alloy (SMA) materials, and dielectric elastomer (DE) materials, as well as eccentric vibration motors. Exciting piezoelectric actuators usually require a relatively high voltage [9, 10, 11, 12, 13, 14, 15]; SMAs exhibit a certain thermal hysteresis [16, 17, 18, 19]; and DE actuators demand a large electric field, thus posing high requirements on the application environment [20, 21, 22, 23, 24]. Compared with other vibration direct‐drive methods, the eccentric motor‐based vibration direct‐drive achieves the advantages of low excitation voltage, easy control, and easy integration [25, 26, 27, 28, 29, 30, 31]. For example, the monolithic soft robot Leafbot proposed by Linh V N takes an eccentric vibration motor as the vibration source, and it requires no additional transmission components or phase control modules. Its arc‐shaped limb design collaborates with the eccentric motor drive to generate directional motion by virtue of asymmetric friction, enabling the robot to easily cross 70° slopes, a semi‐circular obstacle that is 5 times taller than the body, and rough terrains [25]. Rubenstein et al. developed a representative robot, named Kilobot, actuated by two eccentric motors with a maximum speed of about 10 mm/s, which achieved compact structure, simple actuation, and low cost [26].

Traditional vibration direct‐drive miniature robots with fixed configurations suffer from problems such as fixed structure and single function [25, 27, 28, 29, 30]. For example, robots with fewer legs tend to have stronger steering flexibility, while multi‐legged structures usually possess better payload capacity and obstacle crossing ability. In contrast to conventional miniature robots, modular designs represent a paradigm shift that exhibits greater versatility and adaptability. By disassembling functions such as actuation, sensing, and control into independent and dockable modular units, miniature robots can realize free splicing, reconfiguration, and collaborative work of modules, flexibly integrate the advantages of different configurations, and adapt to the requirements of different operation scenarios on demand. Meanwhile, modular design can simplify the research, development, and integration process of miniature robots, and improve the scalability and robustness of the system—even if some modules fail, tasks can still be completed through reconfiguration and adjustment [34, 35, 36, 37, 38, 39, 40, 41]. However, achieving the synergistic design of modularization and vibration direct‐drive at the miniature scale faces numerous challenges. For example, modular robots often require additional connection mechanisms to switch between different modules, which is not conducive to miniaturization; the vibration direct‐drive mode also places higher stability requirements on the modular connection parts [42, 43, 44, 45, 46, 47, 48, 49, 50]. For instance, the robot system proposed by Li et al. takes disc‐shaped particles with only a single degree of freedom of radial expansion and contraction as the basic modules, and realizes loose coupling between modules through flexible magnetic adsorption. It converts individual random oscillations into collective directional motion by virtue of statistical mechanics effects and achieves collective motion relying on global signals. Although it realizes robust motion with 20% module failure, the connection between its modules has poor stability, and it cannot complete module assembly and configuration adjustment [47]. Gao et al. proposed a centimeter‐scale quadruped piezoelectric vibration direct‐drive robot, which adopts a built‐in integrated design to highly integrate the drive, control, and power supply units into a metal body, and realizes the expansion of sensor modules through reserved through‐holes and 3D‐printed connectors, solving the problems of miniaturization and connection stability of module integration under vibration direct‐drive. Nevertheless, its modular expansion adopts a fixed connection mode, which only allows the installation of a single functional module, cannot realize multi‐configuration recombination of drive modules, and lacks the ability of rapid disassembly, assembly, and switching between modules [48].

To meet the demands of centimeter‐scale robots for high‐speed motion, modular design, and strong environmental adaptability, inspired by arthropods, this paper proposes a basic driving unit based on one eccentric motor and adopts a nested locking connection method to realize the configuration expansion of modular robots. This tool‐free, zero‐redundancy nested locking interface completely eliminates extra fasteners that occupy precious internal space, making it a centimeter‐scale vibration‐driven modular platform that achieves multi‐configuration reconfiguration without compromising overall miniaturization. By modulating the number of driving units, the robot can be selectively reconfigured. Integrated compliant silicone connectors are further introduced to isolate inter‐module resonant interference under high‐frequency operation, solving the long‐standing issue of module loosening that plagues all prior vibration‐driven modular systems, which guarantees stable cooperative operation of multiple eccentric motors during long‐term high‐speed locomotion. Fewer driving units yield superior mobility and agility in quadrupedal configurations, while additional units enhance payload capacity and terrain adaptability. The main innovations and contributions of this work are as follows: (i) we propose a modular driving unit utilizing periodic vibration of elastic feet together with a nested splicing expansion scheme. This approach further simplifies mechanical complexity and assembly while overcoming the functional constraints of fixed configurations, thereby endowing the miniature robot with topological reconfigurability and scalable expandability. (ii) a foot‐end optimization scheme suitable for the vibration direct‐drive mode is proposed. Through combined simulation, experimental validation, and systematic comparison of geometries and materials, a chevron‐shaped arrangement is identified that maximizes compatibility with friction‐based vibration actuation. This enables simultaneous high‐speed translation (maximum linear speed of 580.74 mm/s in the quadruped) and agile rotation (maximum rotational speed of 16.64 rad/s). (iii) a highly integrated miniature control system with universal compatibility across configurations is constructed. Synergistic integration of the host computer, control circuitry, and mechanical structure yields a compact robot balancing low mass and small dimensions (58.4 g and 46 mm × 75 mm × 38.8 mm for the quadruped robot), demonstrating efficient motion within a constrained volume. Collectively, the robot employs vibration direct‐drive actuation with eccentric motors under strict miniaturization constraints, achieving efficient energy transmission. Through bionic structural optimization, it further integrates exceptional locomotion capabilities—including high speed and strong obstacle‐crossing ability—within a limited volume, offering a versatile mobile platform for applications such as in‐pipe inspection and environmental sampling in confined spaces.

## 2 Results

### 2.1 Configuration Design and Untethered Actuation for the Modular Robot

#### 2.1.1 Configuration Design of the Driving Unit

**FIGURE 1:**
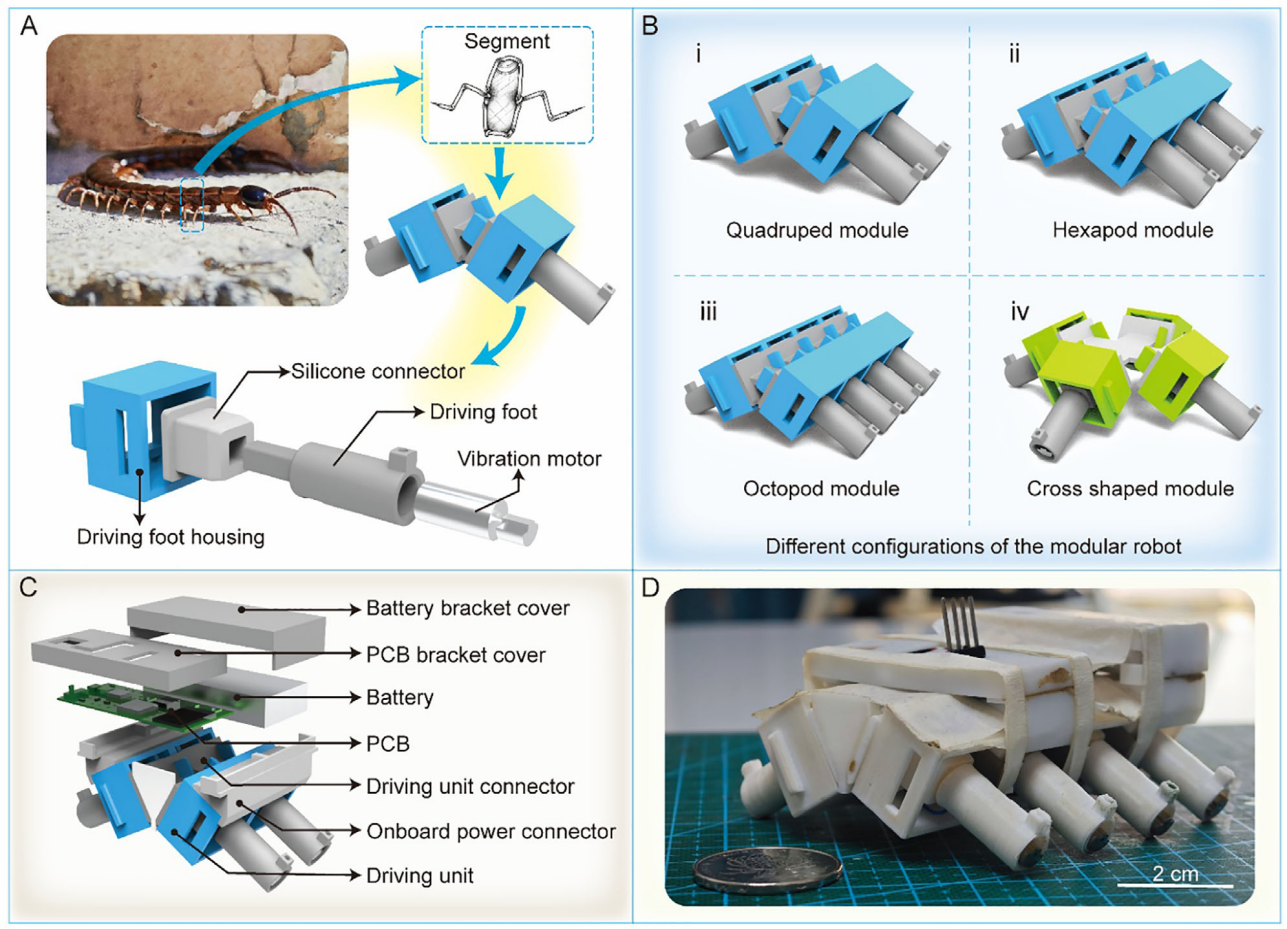
Design and structure of the modular robot. (A) Bioinspired vibration direct driving unit of the modular robot. (B) Different configurations of the modular robot. (C) Exploded view of the quadruped modular robot. (D) The octopod prototype with a coin as a reference (scale bar: 2 cm).

Inspired by the modular and distributed locomotion logic of arthropods, we design a biomimetic modular driving unit and construct a miniature robot. The single driving unit has dimensions of 19 mm × 32.5 mm × 18 mm (see Figure 1A and Movie S1). This driving unit utilizes an eccentric motor as its core power source to provide periodic vibration to the driving feet. The silicone connectors imitate the compliance characteristics of biological joints, while the driving foot housing, equipped with a nested locking mechanism, offers protection for the components and facilitates positioning and connection for the modular design of the robot.

#### 2.1.2 Multi‐Module Assembly Strategies

Based on the modular design strategy, we further extended the assembly configurations of the driving unit and constructed a modular robotic system with multiple morphologies (see Figure 1B). By connecting driving units with various connectors, standardized assembly of these units enables biomimetic morphological reconfiguration. When four driving units are assembled symmetrically, the robot mimics the quadruped gait of mammals or certain arthropods, enabling stable and high‐speed locomotion in relatively flat environments (see Figure 1B(i)). Adding two or four additional driving units to the quadruped module yields hexapod and octopod modules (see Figure 1B(ii, iii), respectively), which emulate the locomotion patterns of multi‐legged arthropods and further enhance the payload capacity and terrain adaptability of the robot. In contrast, when driving units are arranged in a circular arrangement (see Figure 1B(iv)), it renders the robot more flexible and provides additional degrees of freedom for linear motion.

#### 2.1.3 The Integration of the Modular Robot

To achieve more agile and flexible locomotion, this work develops a highly integrated onboard power supply system for the robot. The robot consists of driving units, an onboard power supply, connectors for inter‐module assembly, and an encapsulating shell (see Figure 1C and Movie S1). The onboard power supply employs a high‐discharge lithium‐ion battery integrated with a printed circuit board (PCB), which not only guarantees continuous power delivery but also enables precise regulation of the actuation frequency of the eccentric vibration motors, thus realizing motion control of the robot. Furthermore, connectors for both driving units and the onboard power supply allow rapid docking between actuation modules and the core system. All inter‐module connections adopt mechanical locking and nested structures, enabling assembly and disassembly without specialized tools, thereby facilitating structural expansion and module replacement. Meanwhile, the compliant nature of the silicone connectors further ensures stable force transmission between modules and mitigates structural wear associated with rigid connections. The prototype achieves compactness, miniaturization, lightweight, and reconfigurability (see Figure 1D).

### 2.2 Robot Locomotion Strategies

#### 2.2.1 Driving Strategy of Array Arrangement

**FIGURE 2:**
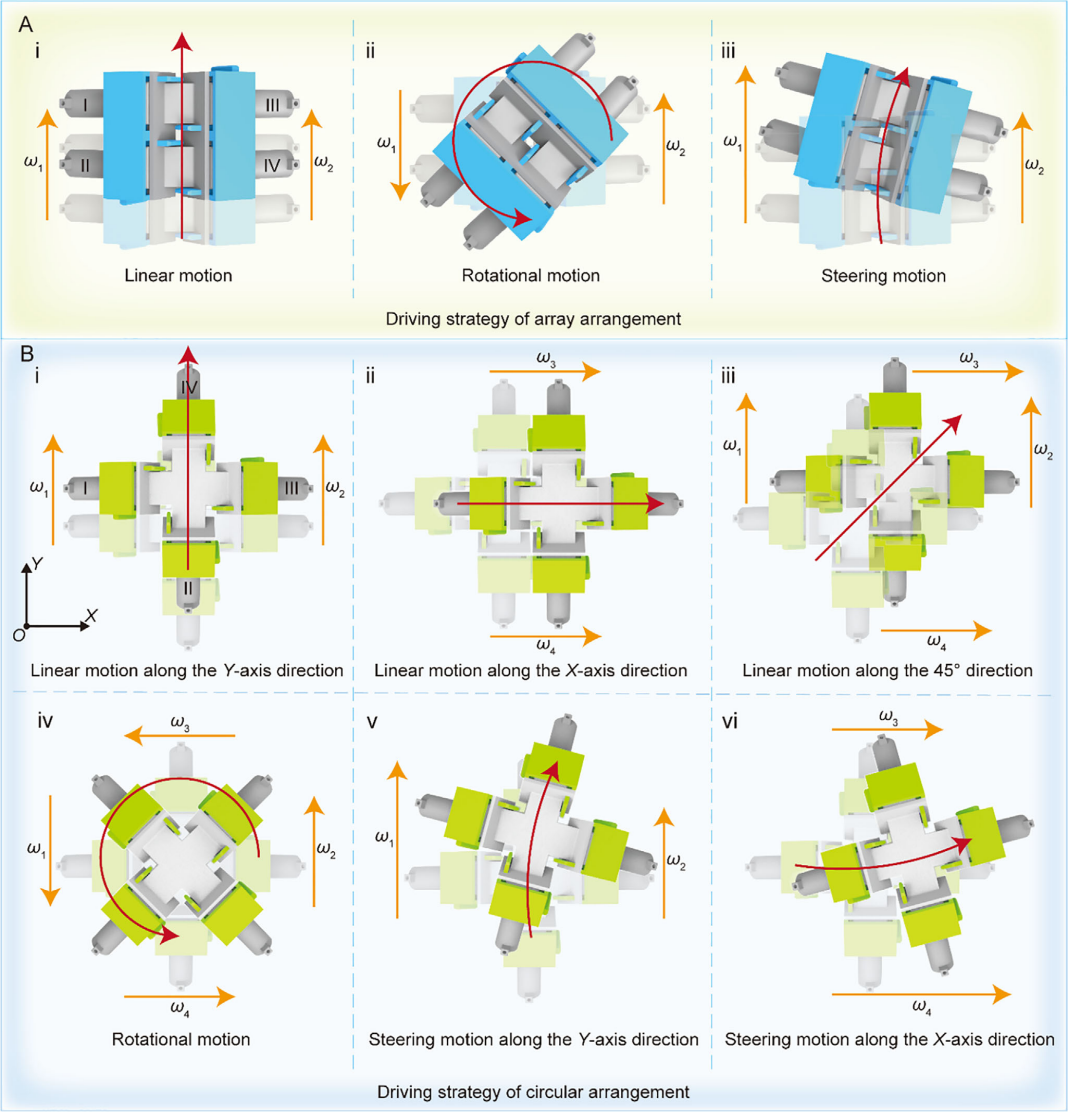
Driving strategy of the modular robot. (A) Driving strategy of the array arrangement. (B) Driving strategy of the circular arrangement.

The driving strategy for the robot with an array arrangement is illustrated in Figure 2A and Movie S2. Each driving unit is actuated by vibration direct drive via an eccentric motor, where the centrifugal force generated by the motor drives the foot to produce periodic friction with the ground. When the eccentric motors of driving feet I and II rotate counterclockwise while those of driving feet III and IV rotate clockwise, the friction forces between the driving feet on both sides and the ground are equal in magnitude and identical in direction, resulting in linear motion of the robot (see Figure 2A(i)). When the eccentric motors of the driving feet on both sides rotate in the same direction, the friction forces between the driving feet and the ground are opposite in direction, inducing rotational motion of the robot (see Figure 2A(ii)). When the friction forces between the driving feet on both sides and the ground are identical in direction but unequal in magnitude, the robot performs a steering motion (see Figure 2A(iii)). The speed of the linear and rotational motions can be tuned by adjusting the excitation voltage (the duty cycle of the PWM excitation signal) of the motors. Similarly, the steering velocity and steering angle of the robot can also be regulated by modifying the motor excitation voltage (duty cycle of the PWM excitation signal).

#### 2.2.2 Driving Strategy of Circular Arrangement

The robot with the circular arrangement enables more flexible motion control through the cooperative regulation of multiple sets of driving units. As shown in Figure 2B(i–iii), linear motions along the *X*‐axis, *Y*‐axis, and 45° oblique direction can be achieved by modulating the vibration states of driving units in different orientations. When the eccentric motors of driving foot II and driving foot IV are excited to rotate counterclockwise and clockwise, respectively, the friction forces between these two driving units and the ground are aligned in the same direction, propelling the robot along the *X*‐axis. By coordinating the driving units in corresponding orientations such that the resultant force points along the 45° direction, the robot can move along this oblique trajectory. When all driving units vibrate in the same direction, the reaction forces of the driving feet generate a continuous rotational torque, enabling the robot to perform in‐place rotation (see Figure 2B(iv)). Furthermore, steering along the *Y*‐axis can be realized by adjusting the vibration frequency difference between driving foot I and driving foot III. Similarly, steering along the *X*‐axis can be achieved by tuning the frequency difference between driving foot II and driving foot IV (see Figure 2B(v, vi).

### 2.3 Locomotion Characteristics of the Modular Robot (External Power Supply)

**FIGURE 3:**
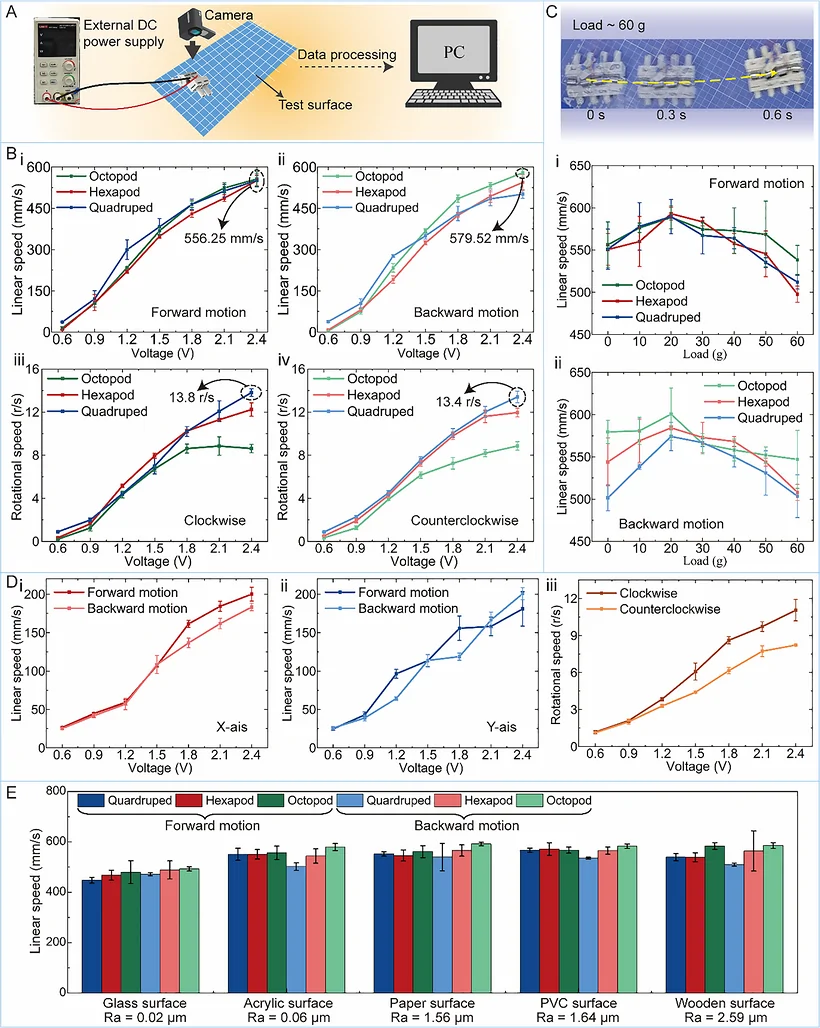
Robotic motion performance testing (external power supply). (A) Testing platform for robotic speed. (B) Linear and Rotational motion characteristics of array‐arranged robots. (C) Load performance of array‐arranged robots. (D) Motion performance of a circular‐arranged robot. (E) Comparison of motion speeds on different surfaces.

We systematically validate the motion performance of the designed modular robot across different configurations, motion modes, and environmental scenarios, with the experimental setup for motion characterization under external power supply (see Figure 3A). The robot is powered by an external DC power supply, and its motion speed is regulated by adjusting the excitation voltage of the power supply. An external camera is used to capture the motion of the robot, and a PC is employed for subsequent data analysis. All motion performance tests are conducted under open‐loop control.

#### 2.3.1 Linear Motion Speed Tests of the Array Arrangement Modules

We test the linear motion speeds of quadruped, hexapod, and octopod modules over an excitation voltage range of 0.6 to 2.4 V. The eccentric motors (operational voltage of 0 to 3.3 V) are limited to 0.6 to 2.4 V to ensure motion initiation and thermal safety: below 0.6 V, the centrifugal force fails to overcome static friction; above 2.4 V, prolonged operation induces coil overheating and accelerates insulation degradation, risking permanent failure.

The results reveal that the motion speed monotonically increased with rising voltage (see Figure 3B(i, ii) and Movie S4). In forward linear motion tests, the octopod module achieves the highest maximum speed of approximately 556.25 mm/s, while the quadruped and hexapod modules also exhibit excellent performance, with maximum speeds of 551.04 and 550.60 mm/s, respectively. In backward linear motion tests, the maximum speeds of the quadruped, hexapod, and octopod modules are 501.57, 544.09, and 579.52 mm/s, respectively. Considering the body lengths of the quadruped, hexapod, and octopod modules (38.8, 58.2, and 77.6 mm, respectively), their maximum relative speeds normalized to body length (BL) are 14.37 BL/s (forward), 9.47 BL/s (forward), and 7.47 BL/s (backward), respectively.

#### 2.3.2 Rotational Motion Speed Tests of the Array Arrangement Modules

Similar to linear motion, the rotational speeds of all modules increase with excitation voltage in the range of 0.6 to 2.4 V (see Figure 3B(iii, iv) and Movie S5). The quadruped module achieves the highest rotational speed, followed by the hexapod module, and the octopod module is the slowest. The maximum clockwise rotational speeds of the quadruped, hexapod, and octopod modules are 13.81, 12.24, and 8.62 r/s, respectively, while their maximum counterclockwise rotational speeds are 13.41, 11.96, and 8.86 r/s, respectively. These results highlight the superior steering agility of the quadruped module.

#### 2.3.3 Load Characteristics of the Array Arrangement Modules

In load characteristics tests, the motion speed first increases and then decreases with increasing payload (see Figure 3C and Movie S6). This trend can be attributed to two competing effects: at low loads (< 20 g), the increased contact force between the driving feet and the ground enhances the propulsive force, boosting speed; at higher payloads, the excitation force from the eccentric motors becomes insufficient to counteract the combined weight of the module and payload, reducing the vibration amplitude of the driving feet and thus decreasing speed. The octopod module maintains a forward speed of approximately 450 mm/s under a 60 g load, while the hexapod module exhibits a more pronounced speed drop, and the quadruped module experiences the most severe speed attenuation at high loads—owing to the octopod module having more eccentric motors, which provide the highest total excitation force. Specifically, when the load exceeds 60 g (which is more than 100% of the self‐weight of the quadruped prototype), the quadruped robot can hardly move.

#### 2.3.4 Motion Speed Tests of the Circular Arrangement Modules

Consistent with the circular arrangement motion strategy analysis, the circularly arranged module possesses multiple degrees of freedom, including linear motion along the *X*‐axis and *Y*‐axis and rotational motion around the *Z*‐axis. Speed test results show that the linear speeds along both axes increase with the excitation voltage of the eccentric motors, reaching maximum forward and backward speeds of 200.21 mm/s (*X*‐axis) and 200.84 mm/s (*Y*‐axis), respectively (see Figure 3D(i, ii) and Movie S4). The rotational speed around the *Z*‐axis also increases with excitation voltage, achieving maximum clockwise and counterclockwise speeds of 11.06 and 8.23 r/s, respectively (see Figure 3D(iii) and Movie S5).

#### 2.3.5 Motion Speed Comparison of the Array Arrangement Module on Different Surfaces

All modules demonstrate adaptability to diverse surface environments (see Figure 3E and Movie S7). When tested on five distinct surfaces with different roughness averages (Ra)—glass (Ra = 0.02 µm), acrylic (Ra = 0.06 µm), paper (Ra = 1.56 µm), PVC (Ra = 1.64 µm), and wood (Ra = 2.59 µm)—the octopod module consistently outperforms the others in both forward and backward linear speeds across all scenarios, followed by the hexapod module. The quadruped module exhibits inferior speed on low‐friction surfaces such as paper. Overall, the octopod module, with its greater number of actuation units, exhibits superior motion stability and speed performance in complex environments.

### 2.4 Locomotion Characteristics of the Modular Robot (Onboard Power Supply)

**FIGURE 4:**
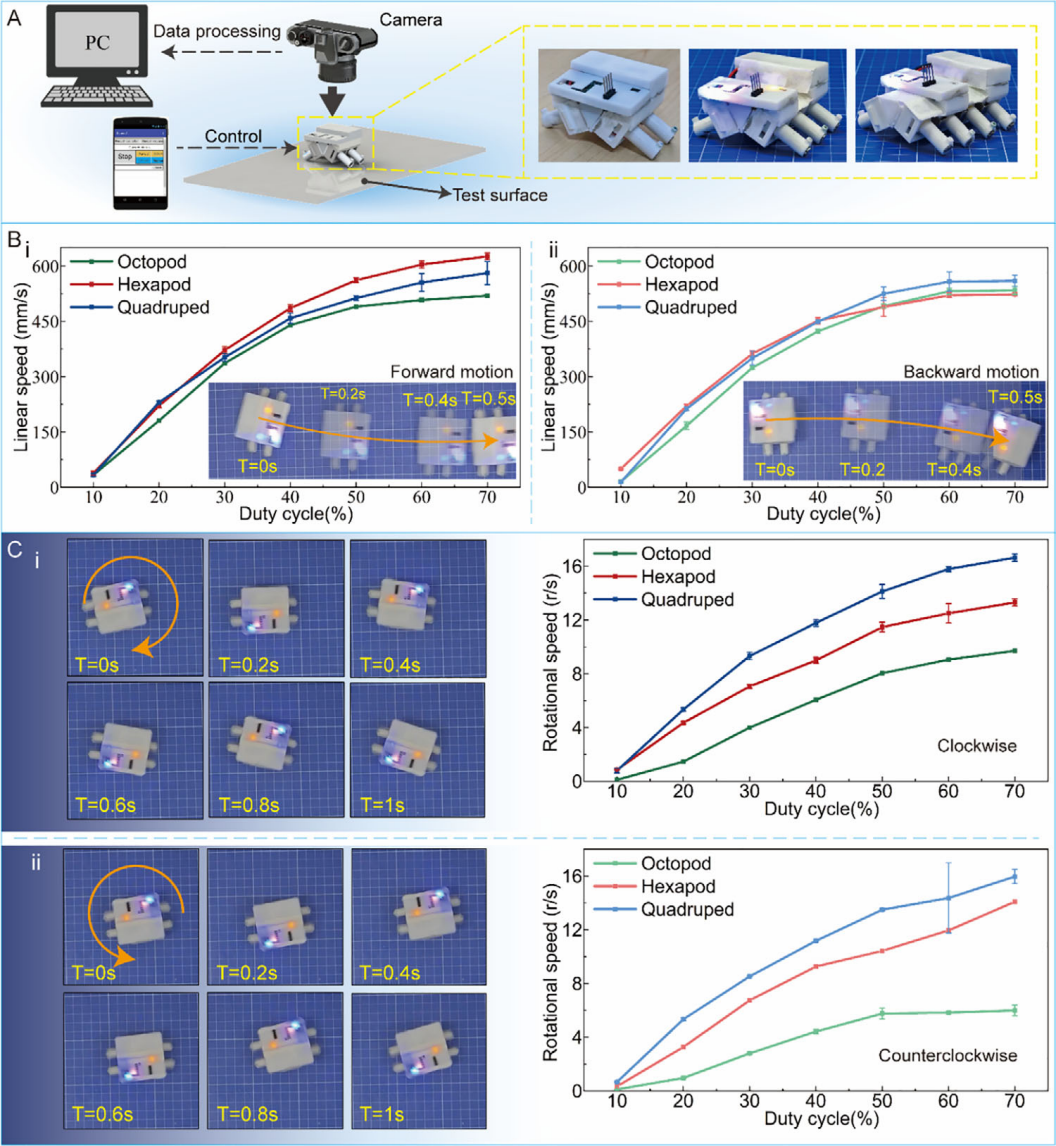
Robotic motion performance testing (onboard power supply). (A) Testing platform for the modular robots. (B) Linear motion characteristics of the modular robots. (C) Rotational motion characteristics of the modular robots.

In the test of the motion characteristics with an onboard power supply, the robot is controlled via the developed smartphone‐based host computer, with motion captured by a camera and analyzed on a PC (see Figure 4A). Under the onboard power supply condition, the supply voltage of the driver chip is 3.3 V. The corresponding relationship between the duty cycle and the effective excitation voltage is 10% duty cycle corresponding to 0.33 V. 73% duty cycle corresponds to 2.4 V. This range completely covers the 0.6 to 2.4 V voltage range of the external power supply test. All tests are performed under open‐loop control.

#### 2.4.1 Linear Motion Speed Tests of the Modular Robots

The linear motion speed exhibits a similar increasing trend with voltage, regulated by adjusting the duty cycle of the PWM signal to modulate the excitation voltage of the eccentric motors. The linear speed increases monotonically with the PWM duty cycle (see Figure 4B(i, ii) and Movie S8). In forward motion, the hexapod robot achieves the highest speed, followed by the quadruped robot, and the octopod robot is the slowest. The maximum forward linear speeds of the quadruped, hexapod, and octopod robots are 580.74, 626.04, and 519.64 mm/s, respectively, while their maximum backward linear speeds are 559.56, 522.88, and 533.74 mm/s, respectively. Notably, the linear speeds under the onboard power supply are higher than those under the external power supply, demonstrating the advantage of the untethered actuation scheme.

#### 2.4.2 Rotational Motion Speed Tests of the Modular Robots

The rotational speed increases with the PWM duty cycle (see Figure 4C and Movie S9). The quadruped robot achieves the highest rotational speed in both clockwise and counterclockwise directions, followed by the hexapod robot, with the octopod robot being the slowest. The maximum clockwise rotational speeds of the quadruped, hexapod, and octopod robots are 16.64, 13.30, and 9.72 r/s, respectively, while their maximum counterclockwise rotational speeds are 15.96, 14.09, and 6.00 r/s, respectively. These results confirm the superior rotational agility of the quadruped robot.

### 2.5 System Functionality and Motion Control Experiments

This study conducts a series of system functionality and motion control experiments on the octopod robot with the onboard power supply, comprehensively verifying its motion performance in diverse complex scenarios. The experiments cover obstacle crossing, gully traversal, corrugated pipe navigation, pipeline motion, and specific trajectory tracking, fully demonstrating the adaptability to complex terrains and high‐efficiency motion control capability. The results show that the octopod robot can maintain high‐speed and stable motion in various environments, and its multi‐legged vibration direct‐drive mechanism and onboard power supply system work synergistically to support its completion of complex tasks, laying a foundation for its application in scenarios such as pipeline inspection and narrow‐space exploration. During the system functionality and motion control experiments process, the motions of the robot rely on open‐loop control.

#### 2.5.1 Obstacle Crossing Performance of the Octopod Robot

**FIGURE 5:**
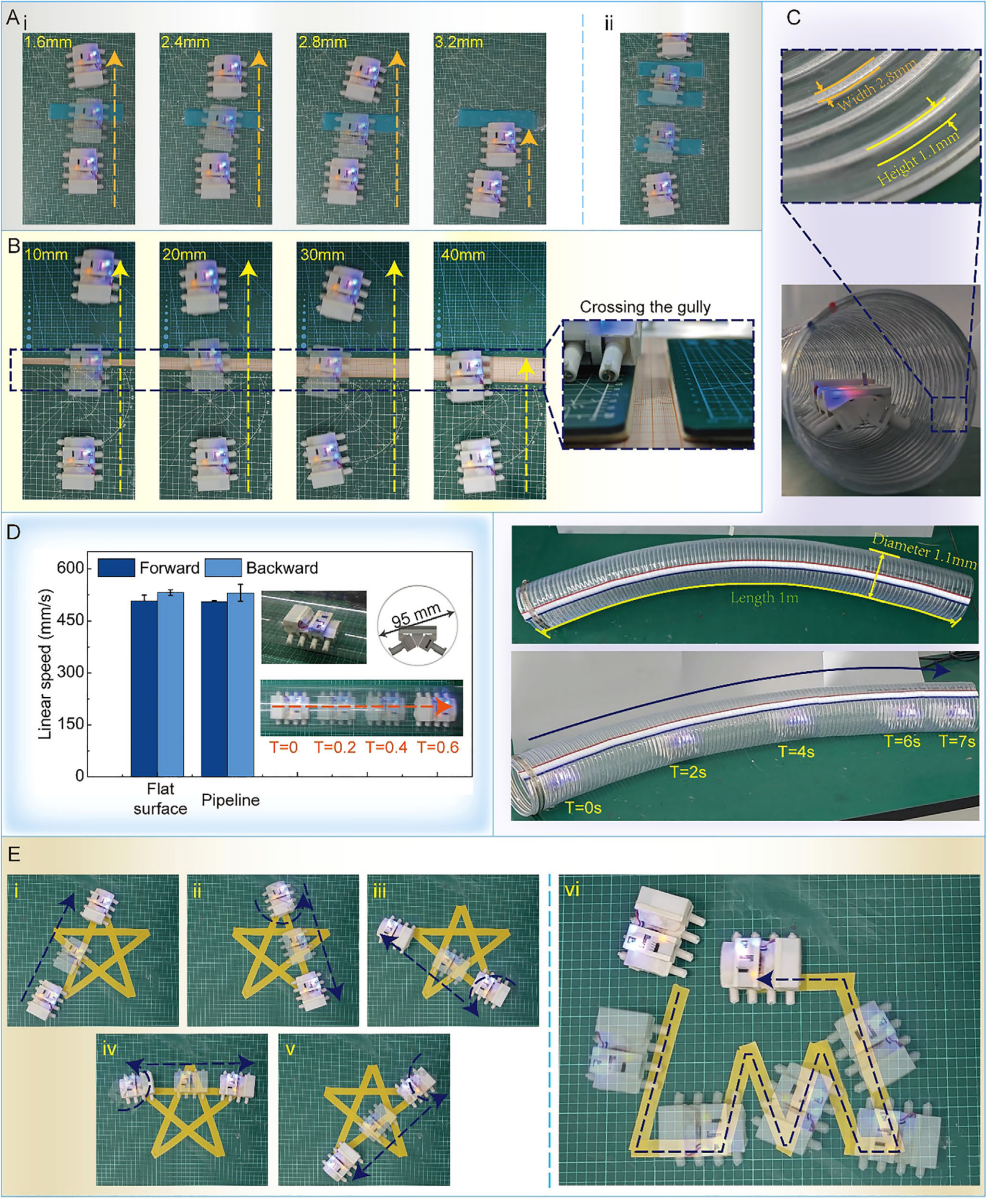
Characteristics and capabilities of the octopod robot (with onboard power supply). (A) Obstacle crossing performance of the octopod robot. (B) Gullies crossing performance of the octopod robot. (C) Corrugated pipes crossing performance of the octopod robot. (D) Pipeline motion performance of the octopod robot and comparison with the flat surface. (E) Trajectory diagram of a robot moving along a specific trajectory.

The octopod robot exhibits convex obstacle crossing capability (see Figure 5A and Movie S10). It can smoothly pass obstacles with heights of 1.6 and 2.4 mm, maintaining stable motion even when crossing the 2.8 mm‐high obstacle. However, when the height of the obstacle is 3.2 mm, the robot cannot pass through the obstacle. This performance is attributed to its multi‐legged structure, which can flexibly adjust the posture of each leg to adapt to the height of the obstacle, and the vibration direct drive mechanism that can generate sufficient instantaneous thrust to overcome the resistance of the obstacle.

#### 2.5.2 Gullies Crossing Performance of the Octopod Robot

The octopod robot demonstrates adaptability to gully terrains (see Figure 5B and Movie S10). It can successfully cross gullies with widths ranging from 10 to 30 mm. When facing a 30mm‐wide gully, it can complete the crossing by coordinating the movement of its multiple legs, adjusting the support points to maintain balance and generate the necessary traction. However, when the width of the gully is 40 mm, the robot cannot pass through the gully. The redundant design of the eight legs allows it to redistribute the load and avoid tipping over in such scenarios.

#### 2.5.3 Corrugated Pipes Crossing Performance of the Octopod Robot

The octopod robot performs well in corrugated pipe environments (see Figure 5C and Movie S10). It can not only traverse the corrugated pipes but also cross the pipe edges. The curvature of the pipe surface does not significantly affect its motion, as the vibration‐driven legs can conform to the pipe's contour, and the multi‐legged synergy ensures continuous propulsion, enabling it to maintain stable motion in this special terrain.

#### 2.5.4 Pipeline Motion Performance of the Octopod Robot and Comparison With the Flat Surface

The linear motion speed of the octopod robot in the pipeline is comparable to that on a flat surface, with both forward and backward speeds approaching 600 mm/s (see Figure 5D and Movie S10). The pipeline is arranged horizontally during the test, which material is acrylic, with a diameter of approximately 95 mm, a wall thickness of 2.08 mm, and a surface roughness of 0.06 µm. This indicates that the onboard power supply can provide adequate power for pipeline motion, and the octopod structure is well‐suited to the confined space of the pipeline. The chevron‐shaped driving feet distributed around the body make full contact with the inner wall of the pipeline, converting the vibration thrust into efficient linear motion, with minor speed loss compared to flat surface motion.

#### 2.5.5 Trajectory Diagram of a Robot Moving Along a Specific Trajectory

The octopod robot can accurately complete complex trajectory movements (see Figure 5E and Movie S10). It can precisely follow preset trajectories such as a pentagram trajectory and a trajectory in the form of LAAT characters. By adjusting the vibration phase and amplitude of each driving unit, it can achieve turning, accelerating, and decelerating operations, demonstrating excellent trajectory tracking ability and motion flexibility. This capability makes it suitable for tasks that require precise path planning in complex environments.

## 3 Discussion and Conclusion

### 3.1 Vibration Direct‐Drive Actuation

The eccentric‐motor‐driven vibration direct‐drive scheme implemented in this study provides a highly effective actuation solution for centimeter‐scale modular robots. By directly coupling centrifugal inertia to substrate friction, this architecture eliminates gearboxes, bearings, steering mechanisms, and multi‐degree‐of‐freedom joints, thereby removing transmission backlash, frictional losses, and assembly complexity inherent to electromagnetic wheeled or multi‐jointed legged systems. Structural resonance is harnessed to amplify energy transfer efficiency, yielding compact, robust locomotion while substantially enhancing endurance and payload capacity. These attributes align directly with the ongoing drive toward miniaturized, lightweight, high‐power‐density mobile robots.

Eccentric motors offer several practical advantages over piezoelectric, SMA, or DE actuators commonly used in vibration direct‐drive miniature robots. Operating at low voltages (0–3 V) with straightforward open‐loop PWM control, they avoid the high‐voltage requirements, thermal hysteresis, or stringent environmental constraints of smart‐material, while enabling stable, multi‐module coordination without phase‐tuning electronics. A single motor per unit suffices to generate directional propulsion through asymmetric friction, circumventing the multi‐actuator synchronization challenges typical of legged microrobots and the slippage issues of wheeled designs at the microscale. Collectively, these features make eccentric‐motor‐driven vibration direct‐drive a highly suitable and practical actuation strategy for untethered centimeter‐scale robotic systems.

### 3.2 Optimization of Robotic Driving Foot‐End Configurations

Locomotion efficiency in vibration direct‐drive robots hinges on actuator–substrate coupling. Systematic iteration—from rigid pin feet to arch‐shaped, foam, and finally chevron‐shaped footless designs—revealed that minimal mass and kinematic rectification, rather than complex appendages, maximize propulsive output. This structural simplification reduces energy dissipation and transmission delays, enabling the quadruped robot to reach 580.74 mm/s linear speed and 16.64 rad/s rotational speed—performance metrics that surpass many fixed‐configuration vibration direct‐drive robots of comparable scale.

### 3.3 Modular Design of the Robot

Arthropod‐inspired modularity breaks the inherent function limitation of conventional fixed‐configuration vibration‐driven miniature robots. We propose tool‐free nested locking interfaces on the standardized 19 mm × 32.5 mm × 18 mm driving units, which occupy almost no extra redundant space and allow the robot to be quickly reassembled into four distinct morphologies: quadruped, hexapod, octopod and circular configurations without using any specialized tools. The integrated compliant silicone connectors not only achieve effective vibration isolation between adjacent driving units to eliminate resonance interference under high operating frequencies, but also maintain long‐term structural stability under continuous high‐intensity vibration impact, solving the long‐standing loosening problem that plagues previous modular vibration‐driven systems. This modular topological design realizes explicit task‐specific performance optimization: quadruped configurations with fewer driving units obtain superior agile maneuverability, achieving the maximum rotational speed of 16.64 rad/s, while the octopod configuration with extra driving units delivers outstanding payload capacity and terrain adaptability, sustaining ∼450 mm/s locomotion speed under a 60 g payload. This work circumvents the classic miniaturization and performance trade‐off of existing centimeter‐scale modular vibration‐driven systems, for the first time realizing multi‐morphology reconfiguration without sacrificing the compactness and high‐speed motion performance of the robot, providing a highly versatile platform for various confined‐space operation scenarios such as pipeline inspection and narrow‐area environmental sampling.

### 3.4 Untethered Actuation

Onboard power supply, control, and Bluetooth communication eliminate tether‐induced drag, entanglement, and trajectory perturbations that degrade high‐speed performance. Compared with externally powered modules, untethered operation robots increased linear speed by ∼5.4% and rotational speed by ∼20.5%, while preserving compactness (quadruped robot: 58.4 g, 46 mm × 75 mm × 38.8 mm). The resulting autonomy extends operational range and environmental robustness, as demonstrated by stable traversal of 2.8 mm obstacles, 30 mm gullies, corrugated pipes, and pipelines at speeds approaching 600 mm/s—capabilities that position the platform for real‐world applications in pipeline inspection and narrow‐space exploration.

### 3.5 Limitations and Future Research Focus

Although the current open‐loop system achieves impressive speed, payload, and reconfigurability, closed‐loop control remains essential for precision tasks under variable terrain or payload conditions. Integration of lightweight attitude, infrared, or tactile sensors, together with real‐time feedback algorithms, will enable adaptive gait modulation and obstacle avoidance. Expanding connector designs to support multi‐degree‐of‐freedom or three‐dimensional assemblies could further broaden morphological diversity, while self‐reconfiguration and inter‐robot swarm coordination would unlock collective behaviors for distributed sensing or search‐and‐rescue. Finally, hybrid integration of functional end‐effectors (e.g., grippers or sampling tools) would transform the platform from a pure locomotion module into a versatile operational system.

In summary, this arthropod‐inspired modular vibration direct‐drive robot establishes a minimalist yet high‐performance paradigm that synergizes structural simplicity, resonance‐enhanced efficiency, and on‐demand reconfigurability at the centimeter scale. By overcoming longstanding barriers in transmission complexity, fixed morphology, and tether dependence, the work provides a generalizable foundation for next‐generation miniature robots suited to unstructured and confined environments.

## 4 Methods

### 4.1 Materials of Each Part of the Prototype

All bracket covers and connectors are fabricated via 3D printing using PLA material. The battery selected is a power‐type polymer lithium battery (903048P, Zon. Cell, China) with a capacity of 550 mA. The motor dimensions are Φ6 mm × 17 mm (JCS, China).

### 4.2 The Fabrication and Assembling Process of the Prototype

(i) driving unit fabrication and assembling process: installing the eccentric motor and silicone connector into the driving foot housing; (ii) onboard power supply assembling process: installing the multifunctional PCB and a lithium battery into the onboard power bracket; (iii) modular robot fabrication and assembling process: fix all driving units by nested connections using connectors, and then connect the onboard power supply to the driving units.

### 4.3 Experimental Design and Data Analysis

We separately built robot experimental test systems with an external power supply and an onboard power supply. We select multiple types of test surfaces for speed testing and multi‐surface adaptability testing, such as glass, acrylic, paper, PVC, wooden. The camera (E14, Hikvision, China) is used to capture the motion behaviors of the robot. All speed characteristic test videos are recorded using AMCap and analyzed by Adobe Premiere Pro, and the average speed is calculated by recording the relative positions of the robot at keyframes. In the speed characteristics testing experiment, the error bars on the data points represent the measurement deviations from five repeated experiments. Statistical analysis of the data is conducted using Origin 2018.

## Author Contributions

**Shijing Zhang**: conceptualization, data curation, investigation, writing – review and editing, writing – original draft, funding acquisition, formal analysis, validation, visualization. **Ziteng Liu**: data curation, investigation, validation, formal analysis, writing – original draft, writing – review and editing. **Youwei Liu**: data curation, investigation, validation, formal analysis, writing – original draft, writing – review and editing. **Dehong Wang**: writing – review and editing, formal analysis, funding acquisition. **Yingxiang Liu**: conceptualization, methodology, software, writing – original draft, writing – review and editing, supervision, funding acquisition, project administration, resources.

## Funding

National Natural Science Foundation of China (No. 52225501, No. 52405012, and No. 524B2045).

## Conflicts of Interest

The authors declare no conflicts of interest.

## Supporting information




**Supporting File 1**: advs77834‐sup‐0001‐SuppMat.docx.

**Supporting File 2**: advs77834‐sup‐0002‐MovieS1.mp4.

**Supporting File 3**: advs77834‐sup‐0003‐MovieS2.mp4.

**Supporting File 4**: advs77834‐sup‐0004‐MovieS3.mp4.

**Supporting File 5**: advs77834‐sup‐0005‐MovieS4.mp4

**Supporting File 6**: advs77834‐sup‐0006‐MovieS5.mp4

**Supporting File 7**: advs77834‐sup‐0007‐MovieS6.mp4.

**Supporting File 8**: advs77834‐sup‐0008MovieS7.mp4.

**Supporting File 9**: advs77834‐sup‐0009‐MovieS8.mp4.

**Supporting File 10**: advs77834‐sup‐0010‐MovieS9.mp4.

**Supporting File 11**: advs77834‐sup‐0011‐MovieS10.mp4.

## Data Availability

The data that support the findings of this study are available from the corresponding author upon reasonable request.
