# Supplementary material for: Arthropod‐Inspired Miniature Modular Vibration Direct‐Drive Robot With Untethered Locomotion
Source: Adv Sci (Weinh). 2026 Sep 16:e77834. Online ahead of print. doi: 10.1002/advs.77834 (PMC13583115; doi:10.1002/advs.77834)
Supplement: Supplementary file 1 [file ADVS-9999-e77834-s009.docx]

Supporting Information

**Arthropod-inspired Miniature Modular Vibration Direct-drive Robot with Untethered Locomotion**

*Shijing Zhang#, Ziteng Liu#, Youwei Liu#, Dehong Wang, Yingxiang Liu**

Email: liuyingxiang868@hit.edu.cn (Y.L.).

**The Supporting Information includes:**

**Note S1:** Design of host computer software and onboard power supply design

**Note S2:** Dynamics model of the robot.

**Note S3:** Simulation and configuration optimization.

**Figure S1:** Design of host computer software and onboard power supply design.

**Figure S2:** Dynamics model of the robot.

**Figure S3:** Simulation and configuration optimization.

**Table S1**: Communication protocol design.

**Table S2**: Parameters of the dynamics model.

**Other Supporting Information includes the following:**

Movie S1. Configuration and exploded view of the robot.

Movie S2. Working principle and driving Strategy.

Movie S3. Comparison of robot speeds under different driving foot types (tethered).

Movie S4. Linear motion test of the robot (tethered).

Movie S5. Rotational motion test of the robot (tethered).

Movie S6. Load capacity test of the robot (tethered).

Movie S7. Adaptability for different surfaces test of the robot (tethered).

Movie S8. Linear motion test of the robot (untethered).

Movie S9. Rotational motion test of the robot (untethered).

Movie S10. Experimental demonstration of locomotion performance and environmental adaptability.

Supplementary Note

**Note S1. Design of host computer software and onboard power supply design.**

1.	Host computer software design

The host computer functions of the robot include Bluetooth connection and command sending. To improve portability, the host computer is developed on the Android operating system for mobile phones (see **Fig. S1**, Supplementary Materials). Communication between the host computer and the onboard control system is realized via string transmission and reception. The controller uses 8-bit character strings as transmission commands, with predefined fixed data bits for command recognition. Each command also includes two check bits at both the start and end positions, which reduces the incidence of command misinterpretation in the onboard control system. The host software was developed using the APP Inventor platform, an online open-source Android programming tool developed by Google. The platform enables app development via graphical, block-based drag-and-drop components, which simplifies the development process and supports intuitive design and editing of the user interface.

2.	Onboard power supply and control circuit

The onboard power supply consists of a high-discharge lithium battery for power delivery and a PCB integrating control, communication, and motor driving functions (see **Fig. S1**, Supplementary Materials). A microcontroller serves as the core of the control system, responsible for information processing and system integration. The STM32F103C8T6 microcontroller unit (MCU) is adopted in the control circuit, which satisfies the performance requirements for robotic control. The MCU generates pulse-width modulation (PWM) signals on two output pins to produce variable analog voltages, thereby regulating the rotational speed of a single motor. For untethered communication, a Bluetooth connection is implemented using the HLK-B40 Bluetooth module, which offers advantages including small size, long transmission range, and multi-device connectivity. The motor driver circuit employs the DRV8833 chip, which features high continuous output current, under-voltage protection, over-current protection, over-temperature protection, and short-circuit protection. Additionally, a TPS63020 chip is used in the power conversion circuit to meet the power demand of the on-board system.

**Note S2. Dynamics model of the robot.**

The eccentric motor will exert the excitation force when the eccentric rotor rotates (see **Fig.S2**), and the dynamic model can be obtained as:

$$\omega_{\text{r}}=\text{k}V$$

$$F=m_{\text{r}}r_{\text{r}}\omega_{\text{r}}^{2}$$

The components of the motor excitation force in the horizontal and vertical directions can be obtained as:

$$\left\{\begin{matrix} F_{X}=F\cos(\omega t) \\ F_{Z}=F\sin(\omega t) \end{matrix}\right.\text{ }$$

According to the Hertz contact theory and the Coulomb friction, the contact force and friction force between the driving foot of the robot and the ground can be expressed as:

Due to the inclined arrangement of the driving leg (inclination angle ), the equivalent sliding velocity of the foot can be expressed as:

The term	in the friction model accounts for the geometric bias required for symmetry-breaking, enabling efficient net forward displacement within each vibration cycle.

where kg is the contact stiffness coefficient between the driving foot and the ground, cg is the damping coefficient between the driving foot and the ground, and μ is the coefficient of friction between the robot and the ground.

The dynamic equation of the body and the driving foot can be obtained directly by D'Alembert's principle as:

Due to the presence of the driving foot tilt angle, the horizontal stiffness Kx and vertical stiffness Kz in equations (7) to (10) can be expressed as：

$$\left\{\begin{matrix} k_{\text{x}}=k\cos^{2}\alpha\\ k_{\text{z}}=k\sin^{2}\alpha\end{matrix}\right.$$

Assuming that the steady-state response of equations (8) and (10) is a harmonic motion, it can be derived that:

$$\left\{\begin{matrix} x_{\text{b}}=X_{\text{b}}\cos(\omega t) \\ x_{\text{f}}=X_{\text{f}}\cos(\omega t) \end{matrix}\right.$$

Converting equations (8), (10), and (12) into complex form can be expressed as:

$$-m_{\text{b}}\omega^{2}\bar{X}_{\text{b}}=-k_{\text{x}}(\bar{X}_{\text{b}}-\bar{X}_{\text{f}})$$

$$-m_{\text{f}}\omega^{2}\bar{X}_{\text{f}}=k_{\text{x}}(\bar{X}_{\text{b}}-\bar{X}_{\text{f}})-F_{X}+F_{f}$$

By converting equations (12) and (13), it can be derived that:

$$\bar{X}_{\text{b}}=\frac{k_{\text{x}}}{k_{\text{x}}-m_{\text{b}}\omega^{2}}\bar{X}_{\text{f}}$$

$$\left(\frac{k_{\text{x}}^{2}}{k_{\text{x}}-m_{\text{b}}\omega^{2}}-m_{\text{f}}\omega^{2}\right)\bar{X}_{\text{f}}=-F_{X}+F_{f}$$

The horizontal speed of the driving foot can be expressed as:

$$V_{\text{f}}=\text{j}\omega\bar{X}_{\text{f}}$$

As the maximum horizontal speed of the driving foot is reached, the optimal connection stiffness between the driving foot and the body can be expressed as:

$$k_{\text{x}}^{_{\text{opt}}}=\frac{m_{\text{b}}m_{\text{f}}\omega^{2}}{(m_{\text{b}}+m_{\text{f}})}$$

The model explicitly demonstrates that the chevron-shaped footless configuration (with leg inclination ) achieves kinematic rectification: vertical oscillations of the driving leg are mapped into asymmetric frictional impulses. The time integral of the friction force f over each vibration cycle is nonzero due to the phase difference and geometric bias induced by , producing a substantial net forward thrust. This is the fundamental mechanism responsible for the robot’s superior locomotion speeds at resonance frequencies (200–300 Hz) and its stable bidirectional capability. All model parameters are listed in Table S2.

**Note S3. Simulation and configuration optimization.**

The configuration and material of the driving feet have a significant impact on the locomotion speed of the vibration direct-drive robot. We attempted to construct various foot types, including pin feet, foam feet, specially structured plate feet, and arch feet (see **Fig. S3**A). Among these, the pin feet are made of stainless steel, the foam feet are made of elastic EVA, and the flat feet and arch-shaped feet are 3D-printed using PLA material.

In the modal analysis of the drive module (see **Fig. S3**B), the first-order mode is the first vertical bending vibration of the driving leg at a frequency of 228.43 Hz, and the second-order mode is the first horizontal bending vibration of the driving leg at a frequency of 231.76 Hz. The frequency difference between these two orthogonal directions is only 3.33 Hz, both of which are close to the desired frequency. In the harmonic response analysis (see **Fig. S3**C), the maximum vertical amplitude of the driving leg occurs at 228 Hz with an amplitude of 0.98 mm, while the maximum horizontal amplitude occurs at 232 Hz with an amplitude of 0.92 mm. The peak frequencies correspond to those obtained from the modal simulation. After final design modifications, the dimensions of the driving foot housing are 19 mm × 19 mm × 14 mm, the driving foot dimensions are 9 mm × 11.5 mm × 30.5 mm, and the overall dimensions of the drive module are 19 mm × 32.5 mm × 18 mm. According to simulation results, the bending frequencies lie near the maximum rotational speed of the eccentric motor. When the driving foot vibration stabilizes, the vertical amplitude is approximately 0.28 mm, and the horizontal amplitude is approximately 0.62 mm (see **Fig. S3**D). In transient simulations, the amplitude and motion trajectory were observed at both the housing and the tip of the driving foot. The motion trajectory is larger at the housing than at the foot tip (see **Fig. S3**E), indicating that motion performance likely improves closer to the driving leg. Consequently, we attempted to eliminate the output foot, creating a footless drive module where the driving leg directly propels the robot. By adding connecting components, the footless drive module is positioned obliquely so that the driving leg makes direct contact with the ground. From a front view, the drive modules form a chevron-shaped configuration.

The locomotion speeds of robots equipped with all types of driving feet increased with increasing excitation voltage (see **Fig. S3**F and Supplementary **Movie S3**). The robot with the chevron-shaped configuration achieved a maximum locomotion speed of approximately 501.57 mm/s.

**Fig. S1. Design of host computer software and onboard power supply design.**

**Fig. S2. Dynamics model of the robot.**

**Fig. S3. Simulation and configuration optimization.**

**Table S1: Communication protocol design.**

| Command | Send Message |
| --- | --- |
| Forward | #c00fw0d |
| Backward | #c00bw0d |
| Spin left | #c00sl0d |
| Spin right | #c00sr0d |
| Turn left | #c00tl0d |
| Turn right | #c00tr0d |
| Stop | #cststmd |

**Table S2. Parameter of the dynamics model**

| Parameter description | Symbol |
| --- | --- |
| Body mass of the robot | *M* |
| Body mass in the dynamics model | *m*_b_ |
| Mass of the driving foot | *m*_f_ |
| Mass of the eccentric motor rotor | *m*_r_ |
| Eccentricity distance of the eccentric motor rotor | *r*_r_ |
| Rotation angle of the rotor | *θ* |
| Motor speed | *ω* |
| Excitation force | *F* |
| Horizontal component of excitation force | *F*_x_ |
| Vertical component of excitation force | *F*_z_ |
| Horizontal stiffness coefficient between the driving foot and the body | *k*_x_ |
| Horizontal damping coefficient between the driving foot and the body | *c*_x_ |
| Vertical stiffness coefficient between the driving foot and the body | *k*_z_ |
| Vertical damping coefficient between the driving foot and the body | *c*_z_ |
| Horizontal displacement of the body | *x*_b_ |
| Vertical displacement of the body | *z*_b_ |
| Horizontal displacement of the driving foot | *x*_f_ |
| Vertical displacement of the driving foot | *z*_f_ |
